# Real-time peach detection method in complex environments based on improved YOLOv8 and multi-attention fusion

**DOI:** 10.3389/fpls.2026.1830965

**Published:** 2026-05-07

**Authors:** Shuanghong You, Jinquan Li, Lingyun Yao, Chongxi Yan, Zheng Wu

**Affiliations:** 1 Fruit Research Institute, Chongqing Academy of Agriculture Science, Chongqing, China; 2 College of Engineering and Technology, Southwest University, Chongqing, China

**Keywords:** attention mechanism, deep learning, object detection, peach picking, YOLOv8

## Abstract

Automated peach picking in complex orchard environments faces challenges such as fruit overlap, occlusion by leaves and branches, and low efficiency in continuous localization, which require detection algorithms to achieve both high accuracy and real-time performance. To address these issues, this study proposes a lightweight and high-precision peach detection model named Peach-YOLO based on an improved YOLOv8n framework. First, a Receptive-Field Attention Convolution (RFAConv) module is introduced into the C2f structure of the backbone network to enhance feature representation in salient regions and suppress background interference. Second, a Convolution and Attention Fusion Module (CAFM) is integrated to further strengthen the extraction of key fruit features. In the neck network, a Coordinate Attention-guided high-level screening feature fusion pyramid network (CA-HSFPN) is adopted, which significantly reduces computational complexity while improving semantic representation. Furthermore, the Shape-IoU loss function is introduced to replace the traditional CIoU loss, achieving more accurate bounding box regression through geometric alignment that accounts for object shape. Experimental results on a custom peach dataset show that Peach-YOLO, with a compact model size of only 5.0 MB, achieves a real-time inference speed of 115.7 FPS, an mAP@0.5 of 82.2%, a precision of 78.9%, and a recall of 76.4%. Compared with the baseline YOLOv8n, the mAP, precision, and recall are improved by 3.0, 3.6, and 4.8 percentage points, respectively. Compared with current mainstream detection models, Peach-YOLO demonstrates superior performance in both accuracy and efficiency, providing a lightweight, high-precision, and real-time visual detection solution for automated fruit picking systems.

## 1 Introduction

The Peach plays a vital role in global fruit production, with China contributing around 54.8% of the total cultivation area and 64.1% of global yield in 2021. In China, the peach industry has a total output value of nearly 100 billion yuan (Xu and Chen, 2023). Most peach orchards are located in hilly and mountainous regions, where the complex terrain presents substantial challenges for harvesting operations. Currently, the fruit picking process in these orchards is predominantly manual, with labor for harvesting constituting more than two-thirds of the total workforce required throughout the entire peach production industry (Tang et al., 2020; Diao et al., 2025, Aguiar et al., 2020, Patil et al., 2012; Li, 2012; Biszkup et al., 2024; Ennaji et al., 2023). Efficient and accurate detection of ripening peaches is essential for automated harvesting and the modernization of the peach industry. However, natural orchard environments pose challenges such as occlusion, uneven lighting, fruit overlapping, and variation in size and color (Gao et al., 2018; Erfani et al., 2019; Guo et al., 2024). To overcome these issues, many CNN-based object detection models (Lin et al., 2017) have been developed for fruit recognition.

Recently, the effectiveness of advanced detection models for fruit recognition has been demonstrated in several studies (Yassin et al., 2016; Himel et al., 2024; Hua et al., 2024; Li et al., 2021). Liu and Liu (2024) integrated the Coordinate Attention (CA) into the YOLOv5s model, which enhanced the model’s performance for apple detection under occlusion and different lighting conditions. Yang et al. (2023) proposed an LS-YOLOv8s model for strawberry maturity detection and grading, which is based on the YOLOv8s deep learning algorithm and incorporates the LW-Swin Transformer module, improving the model’s generalization ability via the multi-head self-attention mechanism. Similarly, Lu et al. (2022) introduced a Faster R-CNN variant optimized for grape detection, leveraging deformable convolution to improve performance on irregular shapes and dense clusters. In a more lightweight direction, Zhang et al. (2021) introduced a lightweight fruit detection algorithm for edge devices, based on Light-CSPNet and enhanced with improved feature extraction, downsampling, and fusion modules, enabling real-time detection while maintaining accuracy. Recently, researchers have proposed several lightweight detection models for agricultural applications, such as UAM-YOLO proposed by Wang et al. (2026) for obstacle detection in unmanned agricultural machinery, SD-MSL-YOLO proposed by Yu et al. (2026) for vegetable pot seedling detection, and PPBM-YOLO proposed by Lei et al. (2026) for airborne spore detection in wheat disease cross-infection scenarios. Zhou et al. (2020) developed a real-time kiwi fruit detection model using MobileNetv3 as the backbone, focusing on deployment in edge computing devices. Guo et al. (2025) presented the LR-Inst, a lightweight and robust instance segmentation network, and the proposed model can achieve a detection average precision (AP) of 0.946 and a segmentation AP of 0.944, outperforming several state-of-the-art (SOTA) models. Accurate identification of small target fruits in orchards is also a key concern for researchers. Zhou et al. (2024) proposed a YOLOv5-NMM umbilical orange detection model, which improves the model’s ability to recognize small-sized targets by adding a detection head. Li et al. (2023) proposed a strawberry recognition model named Strawberry Faster R-CNN. This model replaces the feature extraction network of the original Faster R-CNN with a multi-cascade structure and substitutes RoIAlign for RoIPooling, thereby reducing the loss of small-target features during pooling and improving feature extraction capability. For peach fruit detection, Zhao et al. (2023) developed a large peach dataset and proposed a one-stage instance segmentation model, achieving an average accuracy of 72.12%. To enable peach tree detection in complex natural environments, Liu and Yin (2023) developed the Yolov7-peach system, which is designed to recognize immature small yellow peaches. Although the above investigations have improved fruit detection, they mainly focus on single fruit type and neglect factors like occlusion and lighting in real orchard conditions. This Research team (2025) also combined two infield fruit datasets of peaches and strawberries for multiple ripeness stages determination and proposed a lightweight query-based instance segmentation model named FruitQuery, and the proposed method achieved the highest average precision of 67.02 with only 14.08M parameters, outperforming 13 state-of-the-art models with 33 variants. Zhao et al. (2025a, b) proposed a self-supervised method to address two critical challenges: ripeness determination and in-field occlusion. The proposed method is trained in a self-supervised manner on a dataset consisting of fewer than 1% labelled images and the remaining unlabeled images. Moreover, research on honey peach detection remains limited.

In standard CNNs, image features are extracted by computing and summing the dot products between the convolution kernel and equally sized image regions. Despite its effectiveness in computer vision tasks, this method suffers from high computational cost, poor global context modeling, sensitivity to target size variations, and insufficient orientation awareness. In recent years, scholars have proposed more advanced convolution methods. For example, Yu and Koltun (2016) proposed Dilated Convolution, which increases the sensory domain by introducing a dilation rate in the convolution kernel without additional computation. Chollet (2017) proposed the Xception architecture, which uses Depthwise Separable Convolution to decompose traditional convolution into Depthwise Convolution and Point-by-Point Convolution, which greatly reduces the amount of computation in the model. Xia et al. (2022) proposed Deformable Attention, which uses deformable convolution to strengthen the information in focus area. The spatial attention mechanism, by assigning different weights to different spatial locations of the input feature maps, enables the model to focus more on the key regions, thus improving the performance of the model, and becomes an important means that can enhance the performance of convolutional neural networks. Traditional spatial attention mechanisms, such as the Convolutional Block Attention Module (CBAM) proposed by Woo et al. (2018), focus on spatial features: they highlight important regions by weighting information along the spatial dimension while also addressing the problem of parameter sharing in convolutional kernels. YWnet by Liu et al. (2024) further demonstrated the effectiveness of convolutional block attention-based fusion for complex small-target detection. However, the feature maps generated by this type of spatial attention are unable to assign sufficient attention weights to the larger range covered by large-size convolution kernels, thereby limiting the model’s ability to represent complex scenes and large-size targets. To overcome this limitation, researchers have begun to explore other attention paradigms. For example, the works of Liu (2025) and Liu et al. (2025) showed that transformer-driven feature fusion networks and multimodal fusion frameworks based on multilevel feature fusion learning have achieved promising results in multi-label image classification and remote sensing semantic segmentation (Transformer-driven FFN, 2024; Multimodal Fusion, 2024). Against this backdrop, Zhang et al. (2023) proposed Receptive-Field Attention (RFA). Unlike traditional spatial attention, RFA not only focuses on spatial features but also provides sufficient attention weights for large-size convolution kernels, thereby making better use of the contextual information captured by large-size convolution kernels and improving the model’s ability to handle complex scenes and large-scale targets. Based on RFA, researchers further developed RFAConv to replace standard convolution operations, which can significantly improve model performance with a similar computational cost. To address the aforementioned challenges, this study constructs a dedicated dataset of peach images captured in natural orchards and proposes a novel lightweight detection model named Peach-YOLO, based on an enhanced YOLOv8n framework. The core contributions of this work are summarized as follows. First, to improve feature representation for occluded and overlapping fruits, we strategically integrate a Receptive-Field Attention Convolution (RFAConv) module and a Convolution and Attention Fusion Module (CAFM) into the backbone network. Second, the neck is redesigned as a Coordinate Attention-High-Level Screening Feature Fusion Pyramid Network (CA-HSFPN) to enhance multi-scale feature fusion capability while maintaining efficiency. Third, the bounding box regression is refined by replacing the conventional CIoU loss with a Shape-IoU loss for better geometric alignment. Subsequently, we train and evaluate the proposed model on our custom dataset. A comprehensive set of experiments is conducted to validate its performance, including comparisons with the original YOLOv8n and several mainstream models (e.g., YOLOv5n, YOLOv7, YOLOv10n, YOLOv11n, and Faster R-CNN). The experimental analysis focuses on key metrics such as precision, recall, mAP, model size, and inference speed, aiming to verify the effectiveness of our improvements in achieving a balance between high accuracy and real-time performance for robotic peach-picking applications.

## 2 Methods and materials

### 2.1 Dataset acquisition

**Figure 1:**
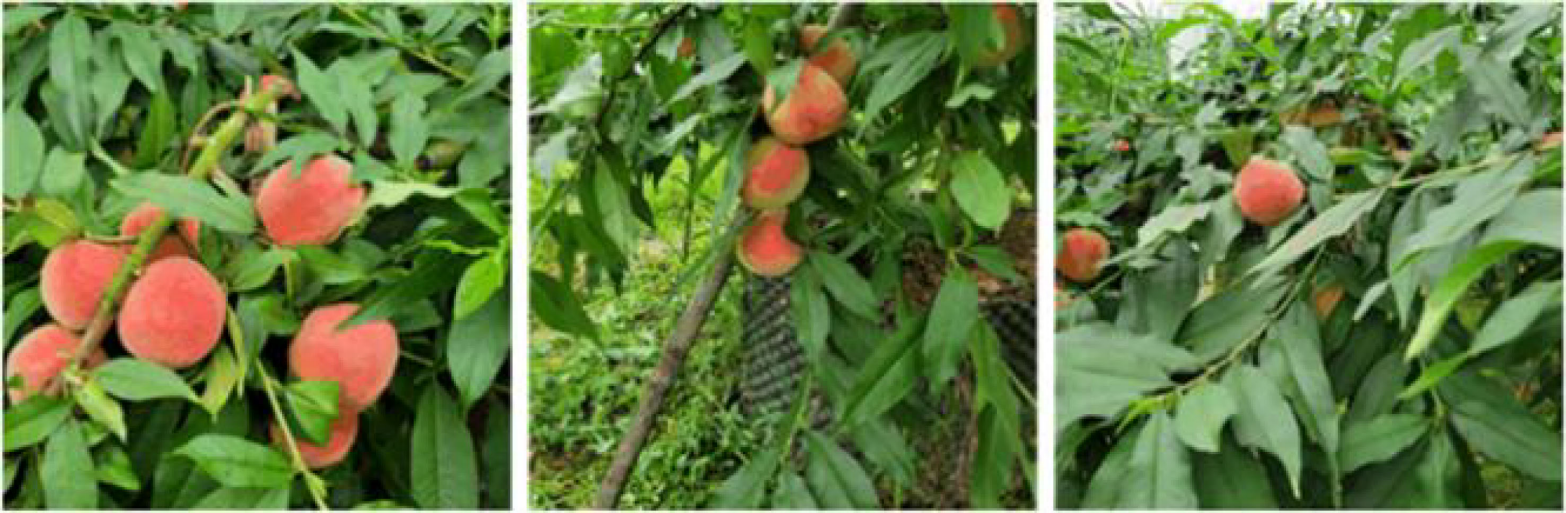
Photo examples of the peach dataset.

There are many varieties of peaches, and they usually ripen from June to September. To ensure that the peach detection model is close to the actual picking conditions and to improve the model’s adaptability to the orchard environment, we chose to capture images in peach orchards during the ripening period. All the peach images used in this paper were collected from the peach plantation area of Chongqing Academy of Agricultural Sciences, including scenes from greenhouse plantations (29°27′14″N, 106°21′57″E) and mountain orchard plantations (29°18′3″N, 106°17′10″E). The images were collected in July 2024, covering different time periods from 9 am to 4 pm. The images were taken from a distance of 40-100 cm from the fruit and from multiple angles under natural light. The equipment used was a Canon EOS M50 Mark II digital camera and an iPhone 15 Pro mobile phone. We chose a 1:1 aspect ratio for both video and photos. The video was recorded at 60 fps and 1440×1440 resolution, resulting in a 10-minute-21-second video. The photos were taken at 3024×3024 resolution, yielding 1210 photos. The photos were screened to eliminate those that were too blurry or did not meet the distance requirement, and 1,178 high-resolution photos were obtained. Frame extraction was performed on the captured video. Because the video was recorded continuously with relatively uniform viewpoint changes, one frame was extracted every 50 frames, yielding 745 photos. The extracted photos were screened to remove those that were too similar or lacked a target, resulting in 620 photos. The two image sets were merged to obtain 1798 high-resolution pictures. The high-resolution photos are rich in semantic information, but they are not suitable for direct use in picking operations and would consume considerable resources if used directly for training. Therefore, the photos were compressed to 640×640 resolution to improve training efficiency while retaining sufficient semantic information. The resulting image dataset is shown in **Figure 1**.

### 2.2 Data labelling and augmentation

This work uses the YOLO-series detection model; therefore, labels were saved in the YOLO-required txt format. If other label formats are needed in subsequent research, conversion will be performed. We used the open-source annotation tool Labeling to label the collected peach images. The Labeling interface is fully functional, supporting VOC and YOLO formats, and each image contains one or more labels. The picking label is ‘Pick’, the leaf covering or overlapping is ‘Covered’, and the branch or trunk covering is ‘Branch’. After annotation, we obtained 1798 labeled photos. During training, the training set is used to learn data features and adjust internal parameters (e.g., weights and biases). The validation set, which is not involved in training, is used to evaluate model performance on unseen data and to optimize hyperparameters. The test set provides the final evaluation of the model’s readiness for real-world tasks. To ensure data independence, the training, validation, and test sets were separated without overlap. The dataset was split into 1,198 training samples, 300 validation samples, and 300 test samples following a 4:1:1 ratio.

**Figure 2:**
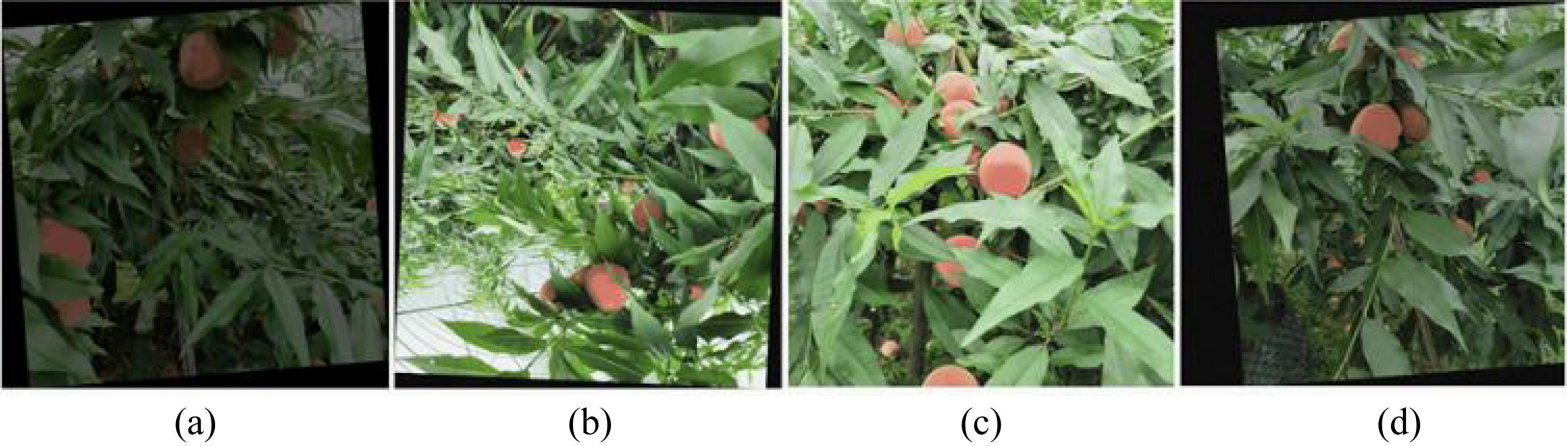
Examples of data augmentation. **(a)** change brightness & Rotation. **(b)** flip & rotate. **(c)** add noise. **(d)** change brightness & panning.

**Figure 3:**
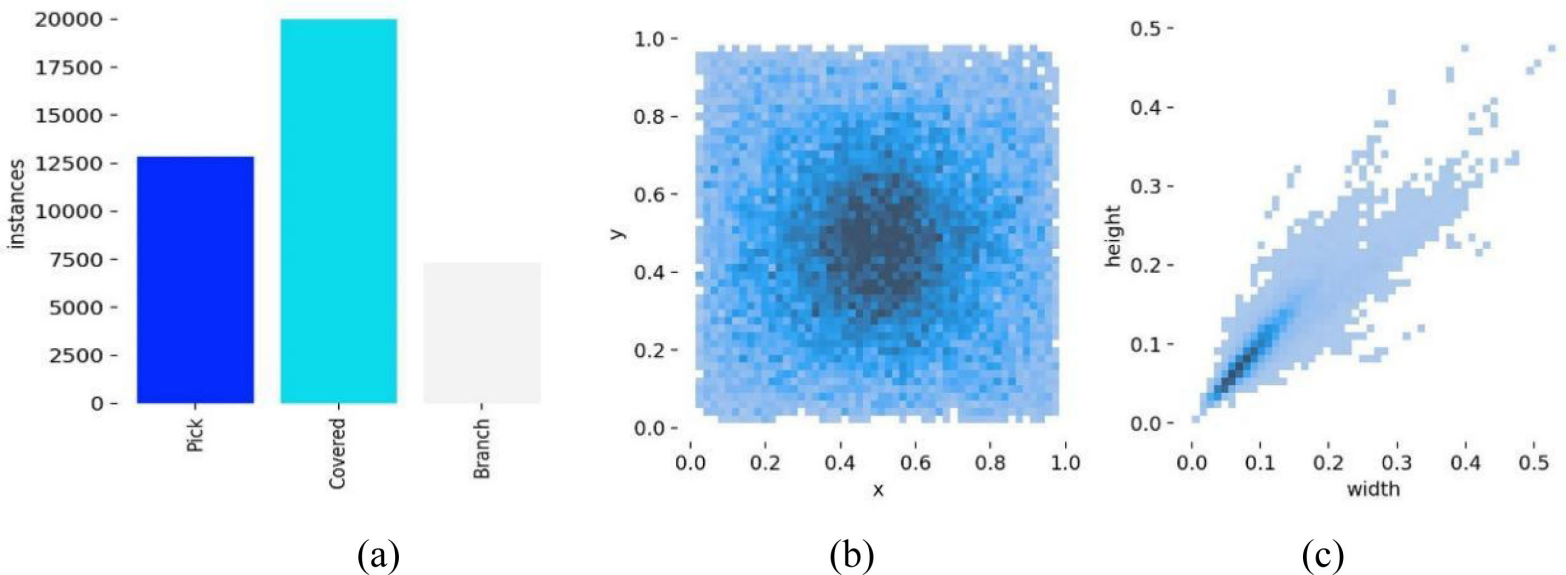
Label statistical diagram. **(a)** label count statistics. **(b)** label position distribution. **(c)** label size distribution.

The photos in the dataset are from a natural orchard environment; although they come from multiple fruit trees, the background is similar and the peach targets resemble each other, which may weaken the model’s generalization. To increase data diversity, enrich the shooting angles and lighting conditions of the training data, and reduce the risk of overfitting, we performed data augmentation on the training set. As shown in **Figure 2**, six random combinations of transformations, such as rotating, adding noise, cropping, changing brightness, flipping, and panning, were performed on the original images, so that the model can adapt to more different viewpoints and target morphologies during training. The expanded training set contains 8386 photos. The number of labels and size distribution of the final dataset are shown in **Figure 3**. The total number of ‘Pick’ tags is 12,831, the number of ‘Covered’ tags is 19,950, and that of ‘Branch’ tags is 7,280.

### 2.3 Peach-YOLO network model

The main focus of this study was to determine whether a fruit is pickable in the presence of occlusion (e.g., shading by leaves or branches). This task requires a target detection model that meets the accuracy and real-time performance required for picking robots. YOLO (You Only Look Once) (Jocher, 2024) is one of the most widely used models for object detection and image segmentation, originally developed by Joseph Redmon and Ali Farhadi at the University of Washington. Owing to its balance of high speed and accuracy, YOLO has gained rapid popularity in both academic research and practical applications. YOLOv8, based on the infrastructure of the predecessor model, solves the traditional anchor frame-based method affected by the quality of the preset anchor frames, adopts the Anchor-Free detection head, which is able to adapt to various sizes of targets and supports tasks such as instance segmentation to meet the needs of more application scenarios.YOLOv8 includes several variants—YOLOv8n, YOLOv8s, YOLOv8m, YOLOv8l, and YOLOv8x—ordered from smallest to largest in network depth and width.

**Figure 4:**
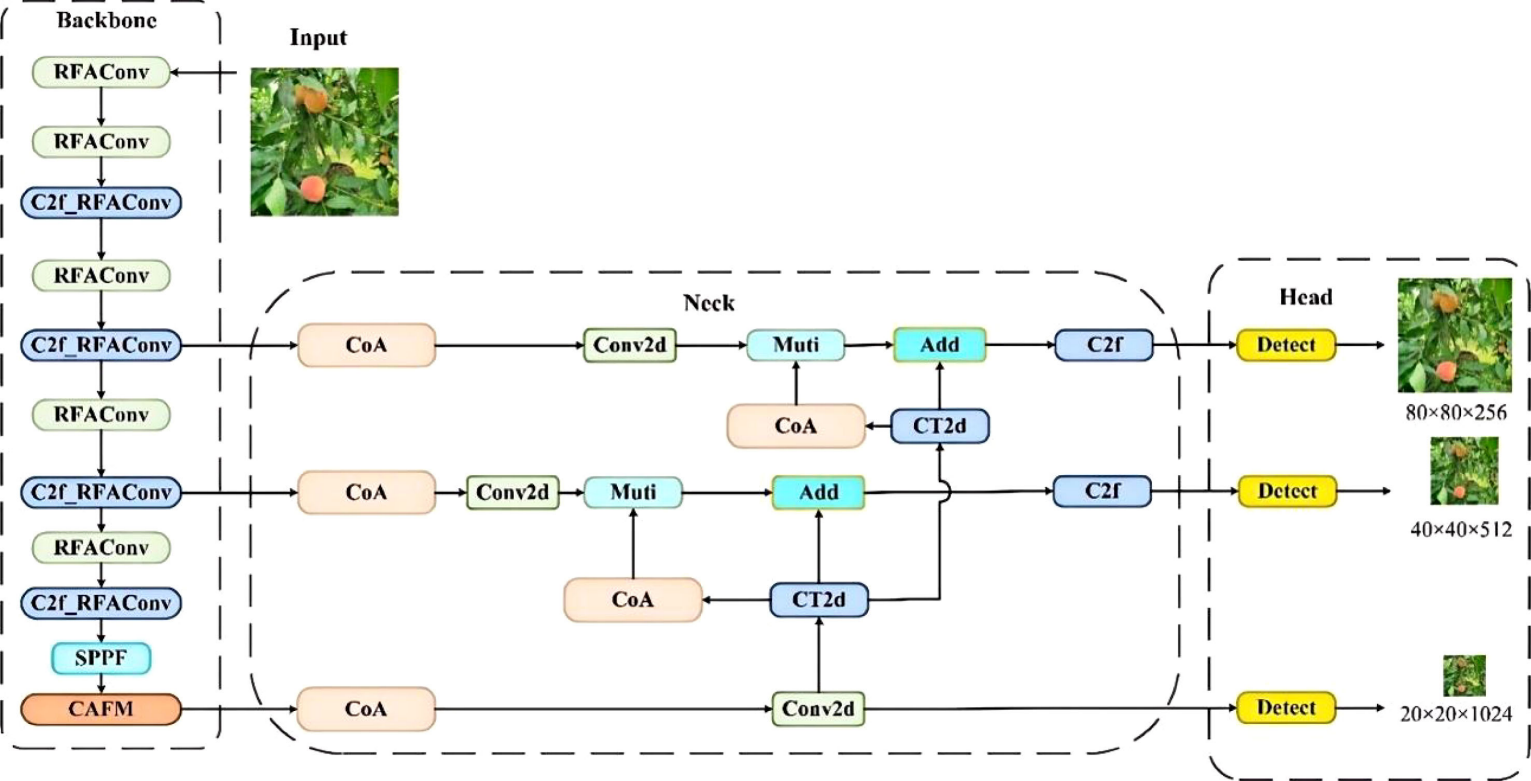
Peach-YOLO model structure diagram.

The test images here were all taken from peach fruits in an outdoor natural orchard environment. Peach trees in natural environments have dense foliage, which makes it very difficult to identify peaches. In addition, peach fruits grow densely, and occlusion among fruits is very common. To further improve the detection model to meet the accuracy and real-time requirements of peach picking, we made the following improvements: (1) Receptive-Field Attention Convolution (RFAConv) (Zhang et al., 2023) is introduced into the backbone network to replace the original standard convolution and improve the feature extraction capability for peach targets of different sizes. (2) A new Convolution and Attention Fusion Module (CAFM) (Hu et al., 2024) is added after the SPPF layer, which combines convolutionally extracted peach features with attention-enhanced peach features to increase the model’s attention to key fruit regions and improve perception. (3) Replace the original neck network with Coordinate Attention-High-Level Screening Feature Fusion Pyramid Network (CA-HSFPN). The coordinate attention mechanism adaptively adjusts channel weights to improve small target detection, and the advanced feature screening pyramid offers stronger feature representation with lower computation. (4) Loss calculation is performed using the Shape-IoU (Zhang and Zhang, 2026) loss function, which obtains the position and shape of the peach bounding box more accurately than ordinary IoU, especially when multi-sized targets coexist. The structure of improved Peach-YOLO model is shown as in **Figure 4**.

#### 2.3.1 Receptive-field attention convolution

**Figure 5:**
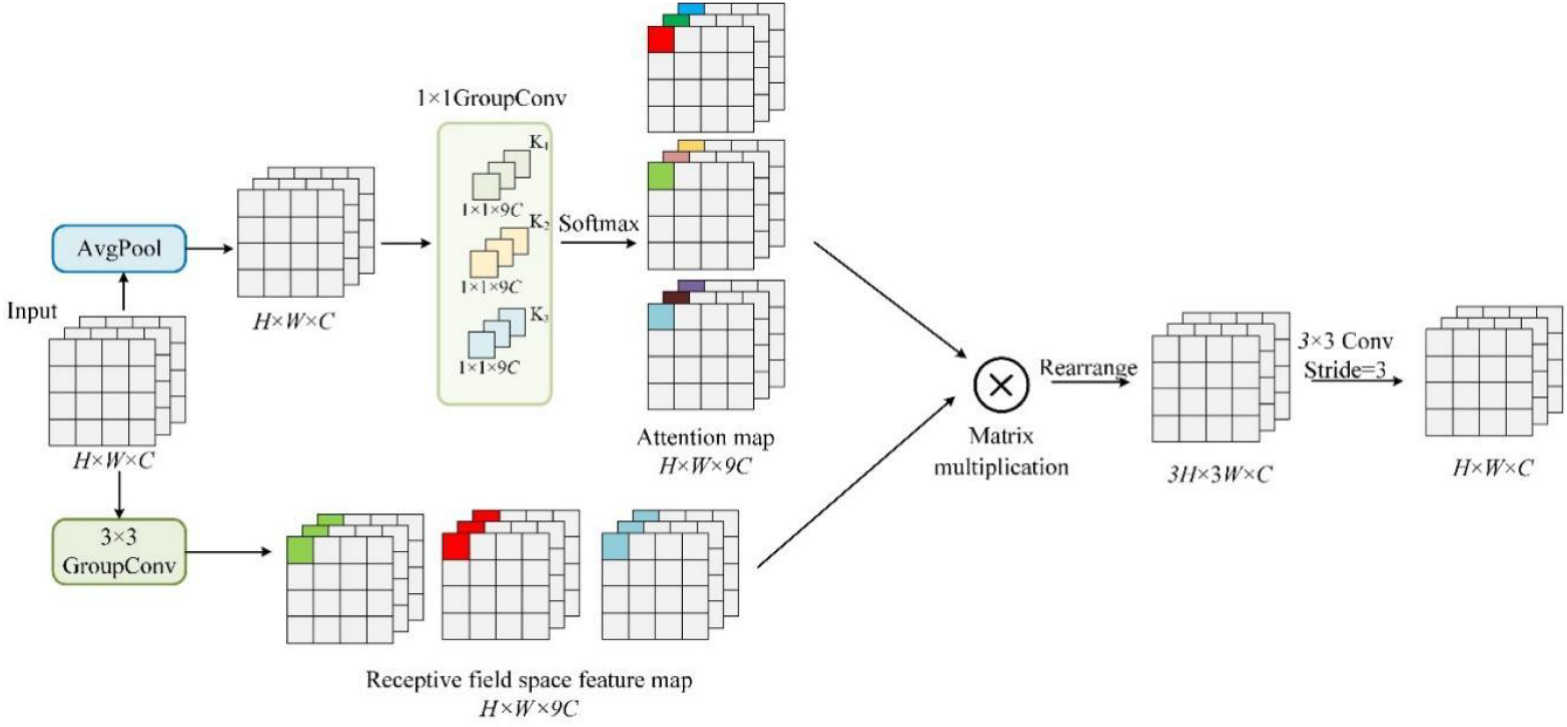
Receptive-field attention convolution.

In this work, to avoid the drawbacks of traditional convolution that affect peach detection performance, RFAConv is introduced into the Conv and C2f modules in the backbone network, and its structure is shown as in **Figure 5**.

RFAConv is divided into an attention acquisition branch and a feature extraction branch. The upper half of **Figure 5** is the attention branch, where the feature information of each receptive field is first fused by average pooling (AvgPool) of the feature map with input dimensions H × W × C. The feature information of each receptive field is then fused by using a convolution kernel of 1 × W and a channel number of 9C. Then the number of channels is increased by using a convolution operation with a convolution kernel of 1 × 1 and a channel number of 9C to achieve the interaction of the information of the sensory domains between different channels, which has a small computational burden and yields an attention map of size H × W × 9C.

The second half of RFAConv is the feature extraction route, which firstly uses grouped convolution with a convolution kernel size of 3×3 and a channel number of 9C to extract features, which has a smaller computational burden compared to the traditional convolution and interacts information from each sensory domain, enabling the model to learn more spatial information. Multiplying the attention map and the spatial feature map, the corresponding attention map can be assigned to each sensory domain feature, so RFAConv can enhance the attention of different features within the sensory domain and prioritize the sensory domain spatial features. The detailed calculation formula is shown in Equation 1.

(1) $$\text{F}=C(\text{Rearrenge}(\text{Softmax}(g^{1\times 1}(\text{AvgPool}(X)))\times \text{ReLU}(\text{Nrom}(g^{3\times 3}(X))))$$

**Figure 6:**
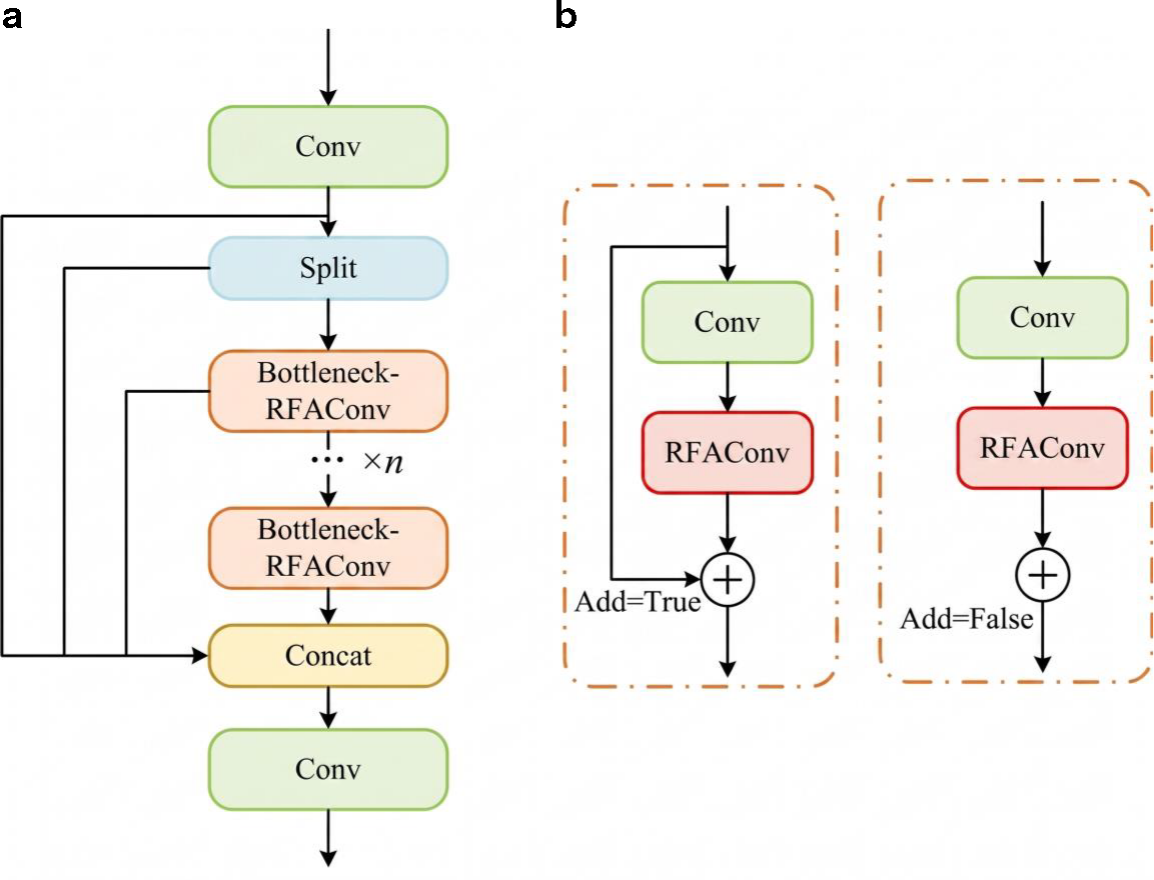
C2f-RFAConv and bottleneck-RFAConv module. **(a)** C2f-RFAConv. **(b)** Bottleneck-RFAConv.

The RFAConv is introduced into C2f in YOLOv8 to form the C2f-RFAConv module and Bottleneck-RFAConv module, the structure is shown as in **Figure 6**.

#### 2.3.2 Convolution and attention fusion module

Conventional convolution relies on a fixed-size convolution kernel to extract the corresponding features, assigning equal weights to all pixel points, and is unable to focus on critical regions. RFAConv alleviates this problem by assigning weights based on feature importance. In the peach detection task, fruit overlap and occlusion are very common, thus requiring the detection model to have reasoning capability. Convolutional operation focuses more on local features and is less capable of extracting global information, and the inference capability is limited (Zheng et al., 2022). Unlike CNN and Long Short-Term Memory (LSTM) (Jin et al., 2021), the Transformer model does not rely on recurrent structure but uses the self-attention mechanism to capture relevant information within the input data, which makes the Transformer perform better in global feature modelling and capturing long-distance dependencies. This makes Transformer better at global feature modelling and capturing long distance dependencies. The core idea of the self-attention mechanism is to be able to consider the dependencies between a certain region of the input data and other regions of the input data when processing that region. This relationship is determined by computing three vectors of queries (Query, Q), keys (Key, K) and values (Value, V) of the input data. These three vectors are not extracted directly from the input data, but are obtained by linear transformation: the query vector is the basis on which the model tries to associate with other elements to obtain dependencies, and is used to match with other key vectors; the key vector is the model’s characterization of the current input element, and each key contains information about the element at that place, and the query vector is used to perform similarity calculations with the key vector in order to determine the element’s weight; the value vector corresponds to the actual content of each element of the input, and the value vector will be calculated with the weight to produce the final output. The specific computation steps are provided in Equations 2–4.

(2) $$\text{Attention}(\text{Q},\text{ K})=\text{Q}\cdot {\text{K}}^{\text{T}}$$

(3) $$\text{Attention Weights}=\text{Softmax}(\text{Q}\cdot {\text{K}}^{\text{T}}/\sqrt{{\text{d}}_{\text{K}}})$$

(4) Output=∑(Attention Weights×V)

where dK denotes the dimension of the key. Through the self-attention mechanism, the output information of each position will fuse the relationship information with other positions.

**Figure 7:**
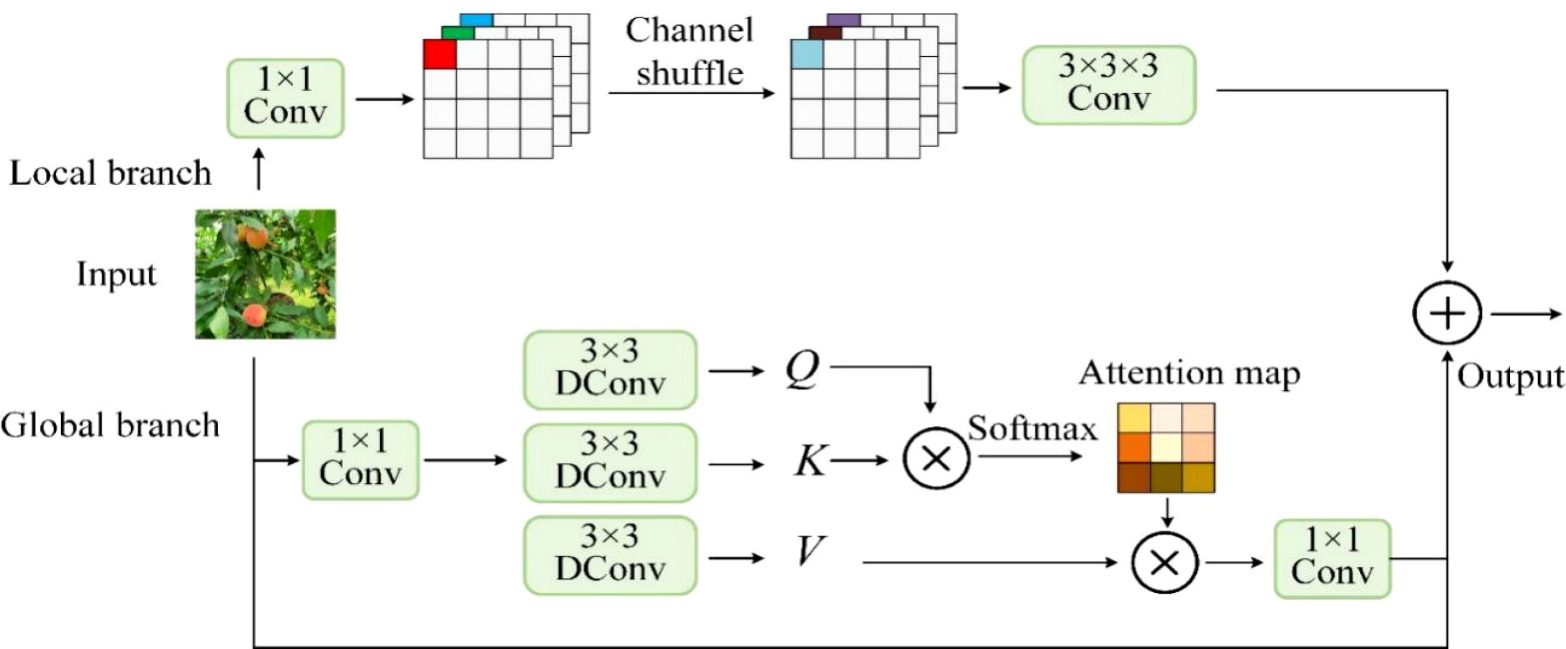
Schematic diagram of CAFM structure.

CAFM combines the local feature extraction ability of convolution with the global modelling of self-attention to enhance the reasoning ability the model during peach detection. Its specific structure is shown in **Figure 7**.

The local branch of CAFM first compresses the feature channels using a convolutional kernel size of 1×1 to retain important information and reduce redundancy. Then the feature channels are rearranged using Channel Shuffle to promote information interaction between different channels to enhance feature diversity and model representation. Finally, a 3×3×3 convolution kernel is used to extract local feature information, which can effectively capture detailed information such as target contours and textures.

The global branch generates Q, K, and V using a 1×1 convolution followed by a 3×3 depthwise separable convolution. This operation helps to map local features to global space for modelling global information. The query vectors and key vectors are reshaped and normalized to obtain the attention map ensuring that the model captures long distance dependencies more efficiently. The value vectors are then weighted and summed according to the attention graph to help the model understand the global structure and contextual information of the entire input image.

In the peach detection task, CAFM extracts fine-grained local features using convolutional operations and channel blending, which can effectively remove noise while retaining detailed information about the target, making it more capable of recognizing occluded or overlapped peach fruits. In addition, through the self-attention mechanism, CAFM can help the model understand the overall structure of the image, which makes it more capable of understanding tasks with very complex backgrounds like the peach picking recognition task. The combination of local and global features enables CAFM to provide richer feature representations for subsequent tasks. This fusion improves the model’s adaptability and prediction accuracy in image processing tasks. CAFM is added to the YOLOv8 network after the SPPF in preparation for the subsequent neck network.

#### 2.3.3 Coordinate attention-enhanced feature fusion pyramid

YOLOv8 uses a feature pyramid network (FPN) as the neck network to perform feature fusion on the multi-scale feature maps extracted by the backbone network, an ability that is particularly important for dealing with objects of varying sizes. Specifically, the FPN extracts and fuses multi-scale features, enabling the model to effectively detect objects of varying sizes. However, FPN also has some limitations. For example, information loss may occur during the fusion of low-level and high-level features, especially in complex backgrounds, leading to ineffective extraction of key features for small targets. Second, FPN is sensitive to the resolution of the input feature maps, and the fusion process may result in the loss of details in low-resolution feature maps, affecting detection accuracy. Although FPN performs cross-scale feature fusion through a pyramid structure, its simple fusion approach cannot fully capture the complex relationships between different scales, resulting in the loss or underutilization of certain fine-grained features. Thus, improving the complementarity and synergy among multi-scale features remains an important research direction.

**Figure 8:**
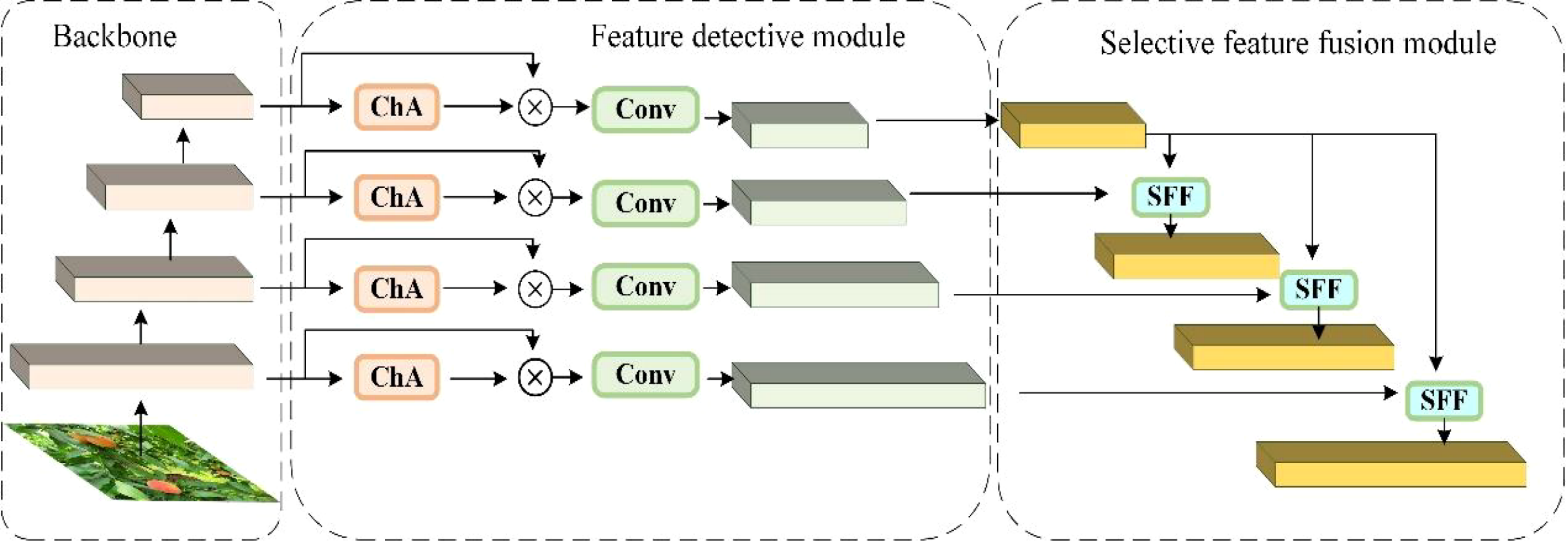
HS-FPN network structure diagram.

An accurate leukocyte detection model was proposed by Chen et al. (2024), based on a Deformable DETR architecture and a multilayer feature fusion network. Feature information at multiple scales was integrated through a High-Level Screening Feature Fusion Pyramid Network (HS-FPN), resulting in improved accuracy for leukocyte target identification. When dealing with the peach dataset, the model faces the same challenge of multi-scale objects and complex backgrounds. To improve model performance on the peach dataset and enhance the representational power of the pyramid network for feature maps of different scales, HS-FPN is used to replace the original YOLOv8 neck network (FPN). Its structure is shown in **Figure 8**. HS-FPN comprises two modules: a feature selection module and a feature fusion module. The feature selection module filters the feature maps extracted by the backbone network to retain salient high-level and low-level information, which is subsequently fused by the feature fusion module.

**Figure 9:**
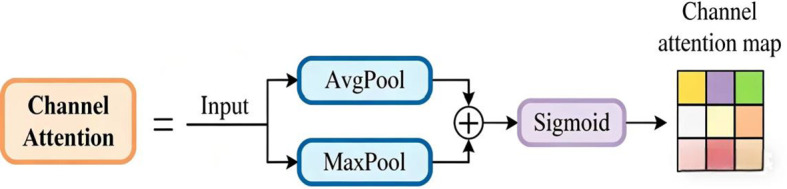
Channel attention mechanism.

In the feature selection module, the input feature map is used to obtain the attention map with the help of Channel Attention (Hu et al., 2018). The structure of channel attention (CA) is shown in **Figure 9**. The inputs undergo average pooling and max pooling separately, the results are summed, and the weight parameters are obtained via the sigmoid activation function. Before feature fusion, the feature selection module performs dimensionality matching of feature maps at different scales, i.e., the feature maps are adjusted to 256 channels using 1×1 convolution.

**Figure 10:**
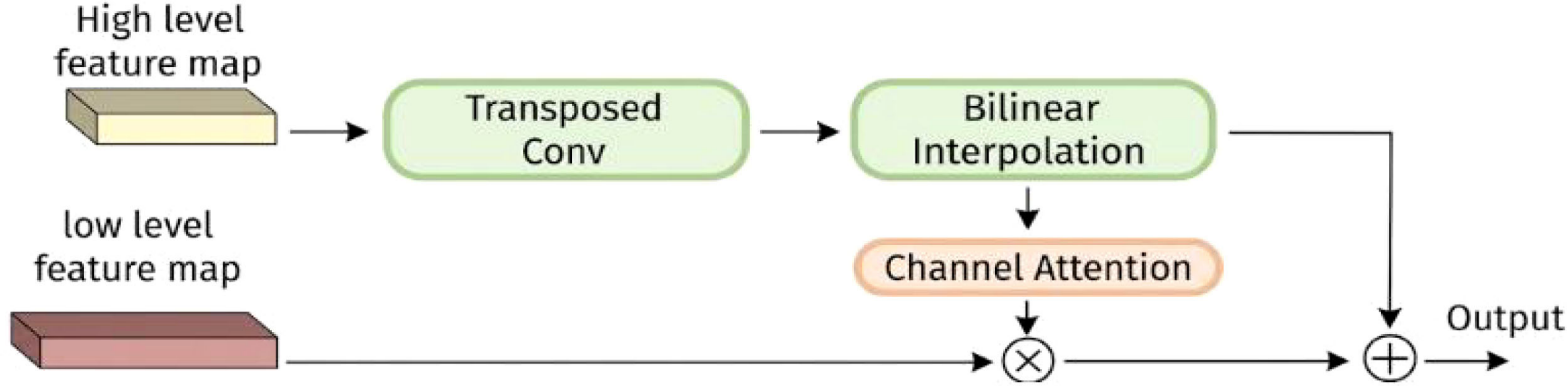
Selective feature fusion.

The feature fusion module of HS-FPN is responsible for fusing the different scale feature maps extracted from the backbone network. The high-level feature maps are rich in semantic information but have low target localization accuracy, while the low-level feature maps have accurate target localization but relatively limited semantic information. The FPN scheme adopts direct element-wise summation of the two types of feature maps, which restricts the acquisition of complex relationships between features. The Selective Feature Fusion (SFF) module uses high-level features as weights to fuse each layer of low-level features. As shown in **Figure 10**, the feature map is expanded by performing a single transpose-convolution operation on the high-level feature map to achieve the feature map, followed by bilinear interpolation for upsampling. Then the high-level features are transformed into corresponding weight parameters by the channel attention module, and then the high-level features are fused with the low-level features, achieving selective feature fusion and enhancing the model’s feature representation capability.

**Figure 11:**
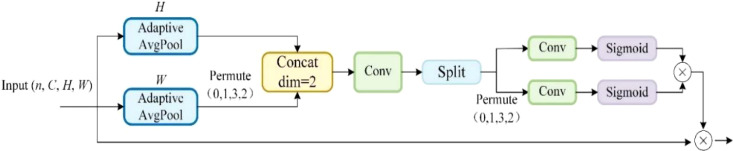
Coordinate attention.

**Figure 12:**
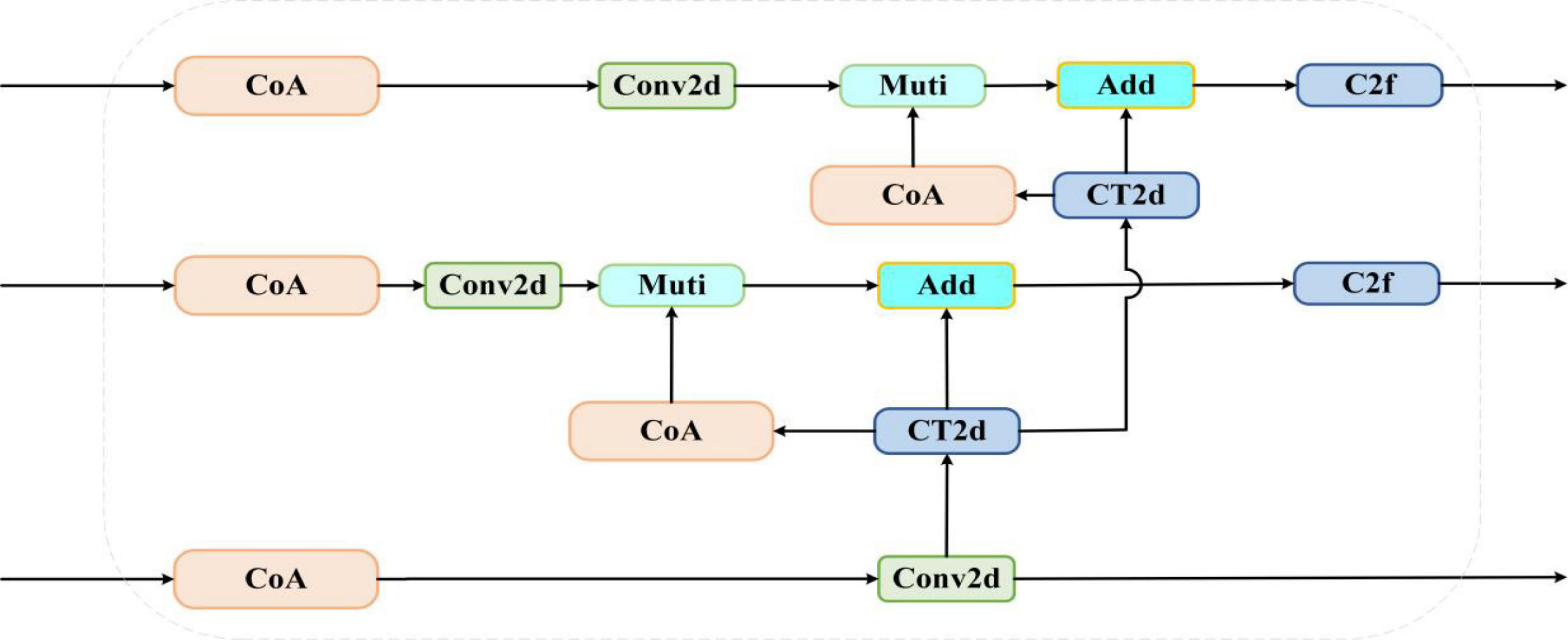
CA-HS-FPN model structure diagram.

The use of channel attention to obtain attention weights in HS-FPN can enhance the feature representation ability of the model. While Channel Attention mainly focuses on the relationship between channels, it pays insufficient attention to the spatial location information in the feature map and usually compresses the spatial dimension information. As a result, it is difficult to achieve high localization accuracy requirements in the peach detection task. To further improve the performance of HS-FPN in peach detection dataset, this paper replaces the Channel Attention used in HS-FPN with Coordinate Attention (CA) (Hou et al., 2021), whose structure is shown in **Figure 11**. For a feature map with input dimensions (n, C, H, W), adaptive mean pooling is performed from the H and W directions, respectively. Adaptive average pooling can pool feature maps of any size in the direction given by the parameters. This not only reduces the size sensitivity of the model but also compresses the features in the corresponding directions to reduce the noise effect. After adaptive average pooling, a transpose operation is performed on the pooled results in the W direction to obtain a feature map of the same size as the H direction. The results of these two directions are spliced, and then a Conv module is used to encode the spatial information in the H and W directions, followed by a Split operation to split into the two directions (Chen et al., 2021). Convolution and activation are then performed for each of the two directions, after which the results are multiplied to obtain the same number of channels as the input data (Weng et al., 2025). The final weighting is performed with the input to obtain a weighted fusion of the spatial information over the channels (Feng et al., 2024). The CA is added to the HS-FPN to obtain the improved CA-HSFPN, the structure of which is shown in **Figure 12**.

#### 2.3.4 Shape-IoU loss function

In peach detection task, the bounding box regression loss is key to the localization branch, as it directly affects the accuracy of target box localization. Several improved IoU methods have been proposed, but they mainly focus on the relative position between the predicted box and the ground-truth box, ignoring factors such as shape and size (Sun et al., 2019). In the regression task of simple targets, using CIoU can predict the target position and shape more accurately, but CIoU performs poorly with complex target shapes. Shape-IoU can measure the degree of shape similarity between predicted and ground-truth boxes, and in peach detection where peaches are irregularly shaped, Shape-IoU can provide a more accurate matching metric. Therefore, to improve the localization accuracy of the peach target detection model for the target bounding box, Shape-IoU is used in this paper to replace CIoU to calculate the loss, As shown in Equations 5–11.

(5) $$\text{IoU}=|\frac{\text{B}\cap {\text{B}}^{gt}}{\text{B}\cup B^{gt}}|$$

(6) $$ww=\frac{2\times (wgt)scale}{(wgt)scale+(hgt)scale}$$

(7) $$hh=\frac{2\times (hgt)scale}{(wgt)scale+(hgt)scale}$$

(8) $$dis\tan ce^{shape}=hh\times \frac{(xc-xcgt)2}{c2}+ww\times \frac{(yc-ycgt)2}{c2}$$

(9) $$\Omega^{Shape}=\sum_{t=w,\;h}{\left(1-e^{-\omega_{t}}\right)}^{\text{θ}},\;\text{θ}=4$$

(10) $$\left\{ \begin{array}{c} \omega_{w}=hh\times \frac{\left|w-w^{gt}\right|}{max(w,\;w^{gt})}\\ \omega_{h}=ww\times \frac{\left|h-h^{gt}\right|}{max(h,\;h^{gt})} \end{array}\right.$$

(11) $${\text{L}}_{Shape-IoU}=1-\text{IoU}+distance^{shape}+0.5\times \Omega^{Shape}$$

where B denotes the real frame, Bgt denotes the predicted frame, scale is the scale factor, determined by the target scale distribution, and ‘ww’ and ‘hh’ denote the weight coefficients in the W and H directions, respectively, which are related to the shape of predicted frame.

## 3 Experiment analysis

### 3.1 Experimental environment

The experimental platform for training and testing is as follows: the operating system is 64-bit Windows 11, the processor model is 12th Gen Inter(R) Core(TM) i5-12400F 2.5 GHz, the graphics card model is Nvidia GeForce RTX 4060Ti (8G), the RAM is 32GB (3200MHz), deep learning framework is PyTorch 2.0, CUDA version is 11.7, programming platform is PyCharm, and programming language is Python 3.11.

In order to compare the performance of different detection models, all experiments here were conducted in the same environment, and YOLOv8n was selected as the original model for peach leaf reduction disease detection. Based on the hardware performance of test platform, the batch size was set to 16, the number of iterations was set to 500, and other parameters were set according to the model defaults.

### 3.2 Assessment of indicators

To visually compare detection performance, this paper selected precision (P), recall (R), average precision (AP), mean average precision (mAP), and F1 score as the evaluation metrics for detection accuracy. Model size (in MB) is used as an indicator of model complexity. The calculations are shown in Equations 12–16, respectively. In these equations, TP (True Positives) refers to the number of peach instances (label ‘Pick’) correctly detected by the model. FP (False Positives) denotes the number of non-peach instances incorrectly identified as peaches. FN (False Negatives) represents the number of ground-truth peach instances (label ‘Pick’) that were missed. Precision (P) reflects the accuracy of the model in identifying peach targets of the ‘Pick’ class. Recall (R) indicates the model’s ability to correctly detect all relevant targets. Average Precision (AP) represents the area under the Precision-Recall curve and evaluates detection performance for a single category. Mean Average Precision (mAP) is the arithmetic mean of APs across all categories, providing an overall measure in multi-class detection. The F1 score combines precision and recall, offering a more comprehensive evaluation of positive instance identification, overcoming limitations of using precision or recall alone.

(12) $$P=\frac{TP}{TP+FP}$$

(13) $$R=\frac{TP}{TP+FN}$$

(14) $$AP=\int_{0}^{1}P(R)dR\;$$

(15) $$\text{m}AP=1/n\sum_{i=1}^{n}AP_{i}$$

(16) $$\text{F}1=\frac{2*PR}{P+R}$$

### 3.3 Ablation experiment

**Figure 13:**
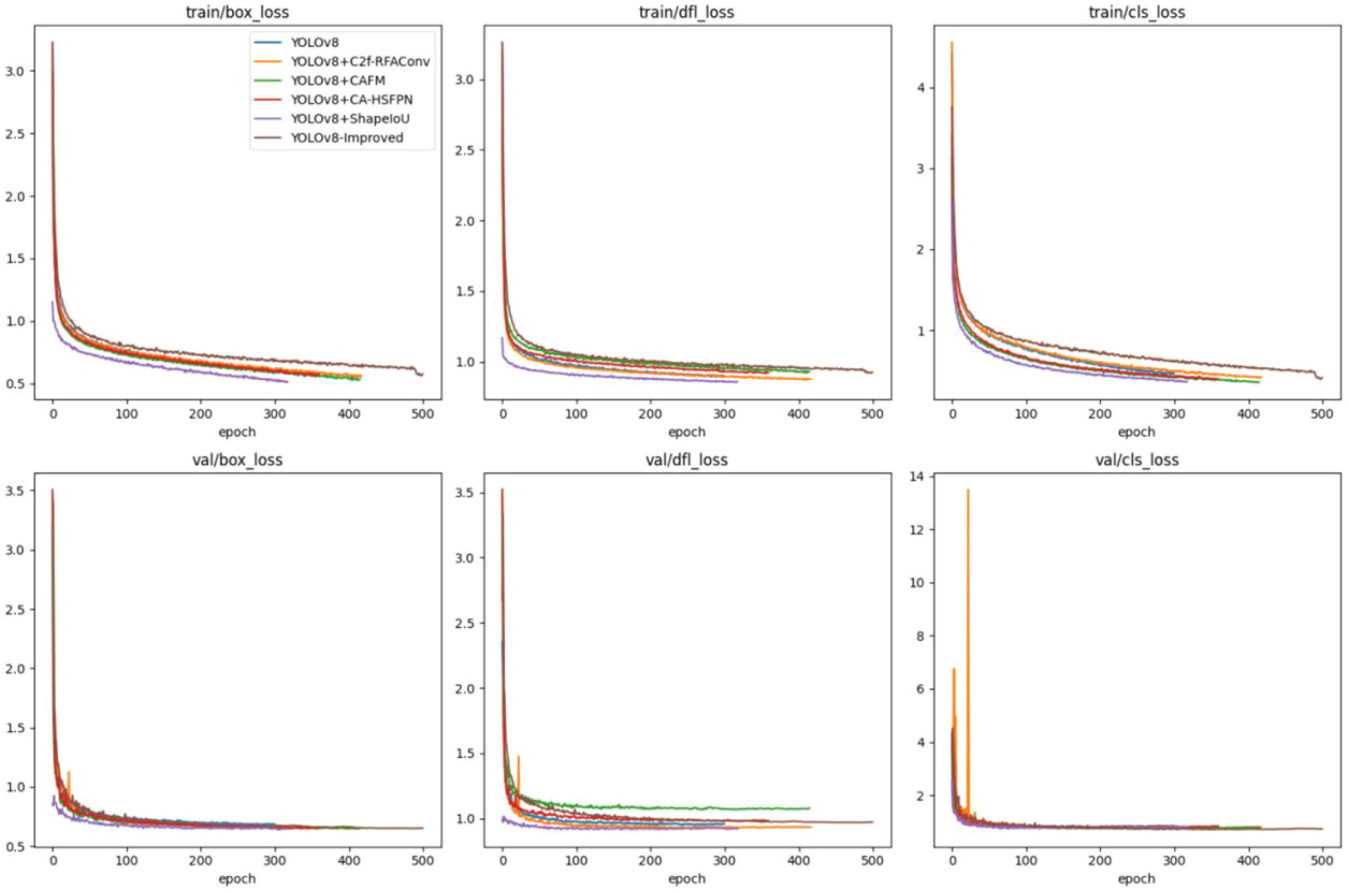
Ablation experiments loss curve comparison.

**Figure 14:**
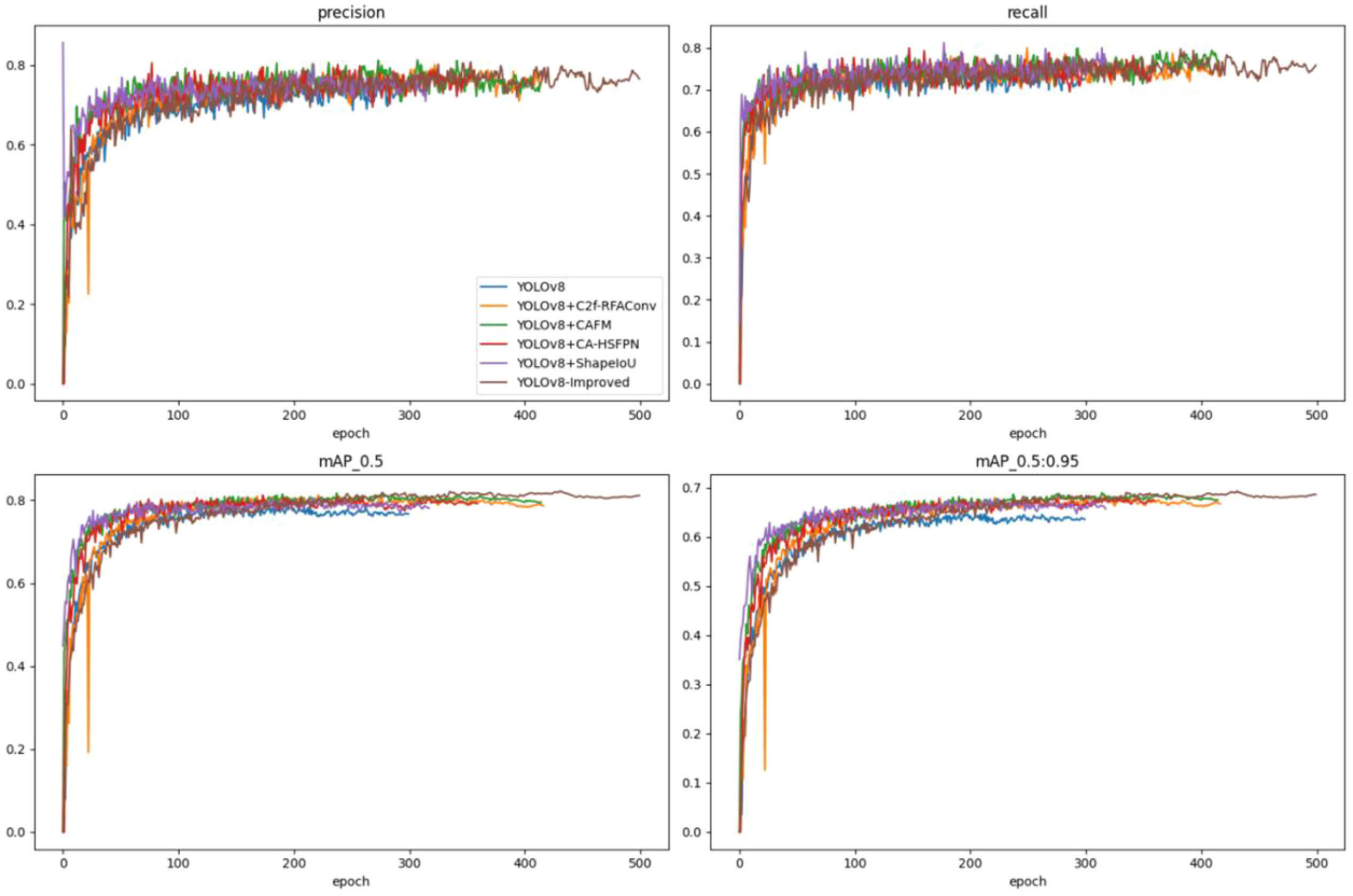
Ablation experiments precision comparison.

To validate the effectiveness of the proposed improvements to the peach target detection model YOLOv8n, ablation experiments were designed as well as to evaluate the effectiveness of each improvement module. A comparison of the model training parameters is shown in **Figures 13** and **14**, while the results of the ablation experiments are presented in **Table 1**.

**Table 1:** Results of ablation experiments.

Models	Precision (%)	Recall (%)	F1	Mean average precision		Model size (MB)	FPS	Pick average precision
				mAP_50_ (%)	mAP_50-95_ (%)			AP/%
YOLOv8n	75.3	71.6	73.4	79.2	66.5	6.3	138.4	82.5
YOLOv8+ RFAConv	78.4	73.3	75.8	80.8	68.3	6.6	129.2	84.4
YOLOv8+ CAFM	77.5	75.5	76.5	81.1	68.9	7.0	118.5	83.8
YOLOv8+ CA-HSFPN	76.1	76.9	76.5	80.7	68.0	4.1	177.1	82.4
YOLOv8+ Shape-IoU	80.4	72.1	76.0	80.6	67.7	6.3	153.8	85.5
YOLOv8 + RFAConv + CAFM	78.2	74.6	76.4	81.4	68.7	6.9	116.5	85.0
YOLOv8 + RFAConv + CAFM + CA-HSFPN	77.9	76.0	76.9	81.8	69.0	5.2	132.0	85.3
YOLOv8 + CAFM + CA-HSFPN	76.9	76.5	76.7	81.2	68.5	4.6	158.0	83.9
Peach-YOLO	78.9	76.4	77.6	82.2	69.2	5.0	115.7	86.0

As shown in the ablation results in **Table 1**, incorporating the RFAConv led to a 3.1% improvement in overall accuracy, a 1.9% increase in average precision for picking target recognition, and a 1.7 percentage point increase in recall. Furthermore, integrating an additional CAFM layer enhanced both the model’s accuracy and recall; however, this improvement came at the cost of increased computational complexity, resulting in a model size increase of 0.7 MB. The lightweight CA-HSFPN increased the model accuracy and recall by 2.2% and 3.9%, respectively, and the model weight was 4.1MB, which was 34.9% lower; after replacing the Shape-IoU loss function, the model accuracy increased by 5.1%, the average precision of the picking target also increased by 3%, and the recall increased by 0.5%, indicating that the model tends to choose higher classification thresholds to reduce misclassification during training, and thus behaves more cautiously.

To further investigate the redundancy and computational bottlenecks introduced by stacking multiple attention modules, three additional group-wise ablation experiments were conducted.

First, combining RFAConv and CAFM (YOLOv8 + RFAConv + CAFM) achieved an mAP50 of 81.4% and a Pick AP of 85.0%, outperforming either single module. However, this combination also resulted in the largest model size (6.9 MB) and the lowest FPS (116.5), indicating clear computational redundancy when two attention modules are stacked without a lightweight neck.

Second, replacing the original neck with CA-HSFPN on top of RFAConv+CAFM (YOLOv8 + RFAConv + CAFM + CA-HSFPN) reduced the model size to 5.2 MB (a 24.6% reduction compared to the 6.9 MB counterpart) and increased FPS to 132.0, while maintaining a high mAP50 of 81.8%. This demonstrates that the lightweight CA-HSFPN effectively alleviates the computational burden caused by multi-attention stacking.

Third, the combination of CAFM and CA-HSFPN (without RFAConv) achieved a model size of only 4.6 MB and an impressive FPS of 158.0, with an mAP50 of 81.2%. This represents a typical “fewer modules + higher efficiency” solution, suitable for scenarios where inference speed is the primary concern.

Comparing these combinations with the full Peach-YOLO (which additionally includes Shape-IoU), the full model achieves the highest mAP50 (82.2%) and Pick AP (86.0%), but at the cost of a moderate reduction in FPS (115.7) and a slight increase in model size (5.0 MB). This trade-off clearly illustrates the balance between accuracy and efficiency: Peach-YOLO prioritizes detection precision for reliable picking operations, while the streamlined combinations (e.g., CAFM+CA-HSFPN) are more suitable for extremely resource-constrained embedded platforms.

The final Peach-YOLO outperformed the initial model by increasing precision and recall by 3.6 and 4.8 percentage points, respectively, boosting the mean average precision (mAP) by 3 percentage points, improving the recognition accuracy of picking labels by 3.5%, and reducing the overall model size by 20.6%. After testing, the improved model achieved an average processing time of 0.0086 seconds per image and a detection speed of 115.7 frames per second (FPS). Although this is slightly lower than that of the initial model, it still meets the real-time requirements of peach picking operations.

As illustrated in **Figure 13**, the model incorporating the improved Shape-IoU loss demonstrates the fastest convergence on the training set and achieves the minimum loss. Although the Peach-YOLO model shows slightly higher loss initially, it eventually achieves the lowest validation loss as the number of iterations increases. In **Figure 14**, a comparison of the improved model’s accuracy, recall, and mean average precision (mAP) is presented. Although the improved model converges more slowly in the initial training phase, it exceeds the original model in mAP after approximately 150 iterations.

Ultimately, the Peach-YOLO model proposed in this study achieved mean accuracy, recall, and average precision of 78.9%, 76.4%, and 82.2%, respectively representing improvements of 3.6, 4.8, and 3 percentage points over the original YOLOv8n model. The detection speed reached 115.7 frames per second (FPS), meeting the real-time requirements of peach-picking robots.

### 3.4 Comparative experiment

To verify the effectiveness of the proposed model, several mainstream target detection models were tested and compared with the Peach-YOLO model. All models were tested on the same dataset under the same environment, and the results are presented in **Table 2**. Comparing **Tables 1** and **2**, it can be observed that, aside from the Peach-YOLO model, YOLOv8n achieves the highest average detection accuracy and average precision, slightly outperforming YOLOv5, YOLOv10, and YOLOv11. In addition, its model size is only 6.3 MB, which supports its selection as the base model in this work. After enhancing the original YOLOv8n model for peach target feature recognition, the proposed Peach-YOLO model achieved an mAP@0.5 that surpasses those of Faster R-CNN, YOLOv3-tiny, YOLOv5n, YOLOv7, YOLOv8n, YOLOv10n, and YOLOv11n by 31.6, 7.7, 3.4, 8.8, 3.0, 3.7, and 4.4 percentage points, respectively. In addition, Peach-YOLO also demonstrates higher precision and recall compared to these baseline models. Meanwhile the Peach-YOLO model weight of 5.0 MB is lower than other mainstream target detection models.

**Table 2:** Comparative experimental results of the Peach-YOLO model with other models.

Models	Precision (%)	Recall (%)	Mean average precision mAP_50_(%)	Model size(MB)	Average precision AP/%		
					Pick	Covered	Branch
Faster R-CNN	61.2	42.2	50.6	108.0	62.2	42.5	41.3
YOLOv3-tiny	74.0	72.2	74.5	16.6	80.4	71.3	70.2
YOLOv5n	73.7	74.6	78.8	5.3	80.0	70.8	68.6
YOLOv7	65.8	71.4	73.4	74.8	70.0	63.7	63.9
YOLOv10n	72.6	74.0	78.5	5.8	80.5	67.5	70.0
YOLOv11n	71.6	74.8	77.8	5.5	79.6	70.1	63.1
Peach-YOLO	78.9	76.4	82.2	5.0	86.0	76.0	74.8

**Figure 15:**
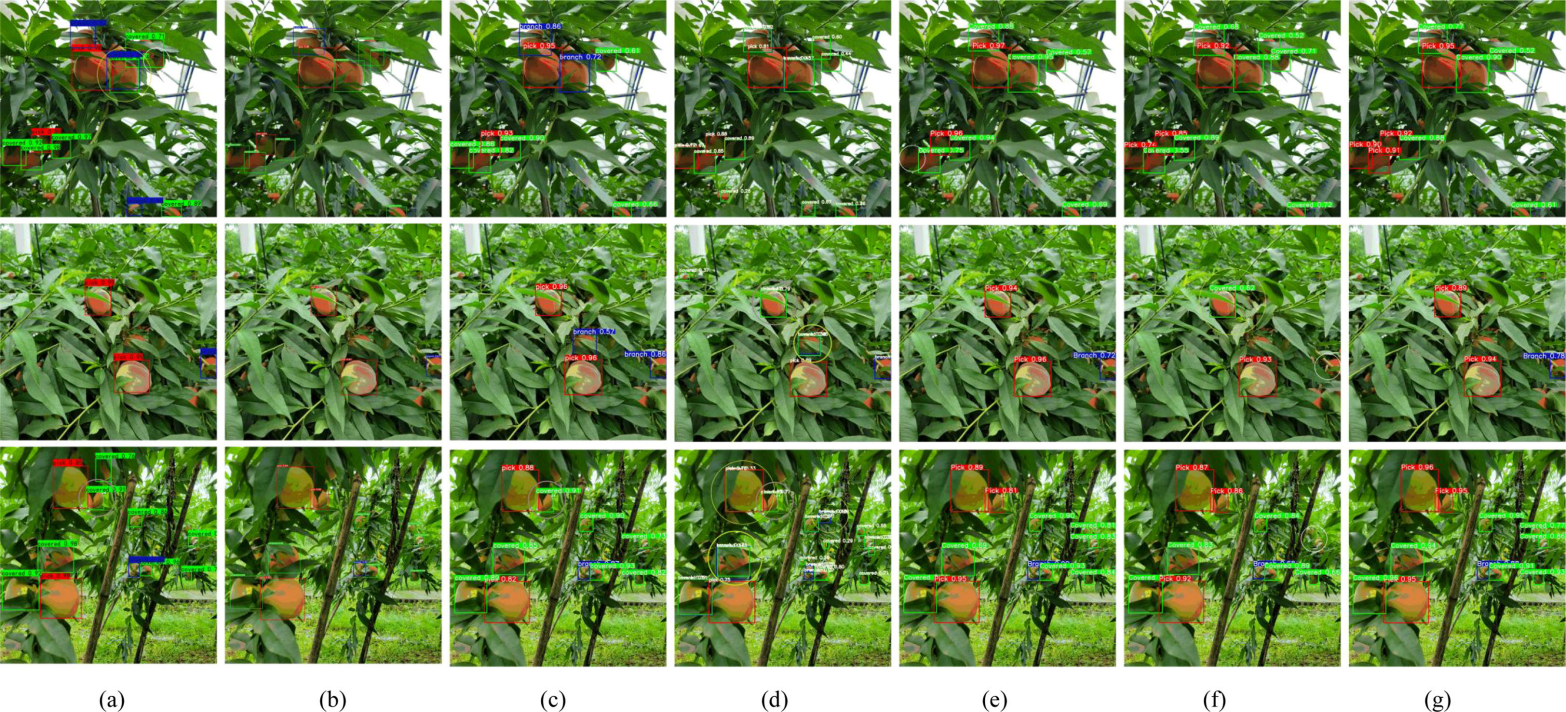
Comparison of test result. **(a)** Faster R-CNN. **(b)** YOLOv3-tiny. **(c)** YOLOv5n. **(d)** YOLOv7. **(e)** YOLOv10n. **(f)** YOLOv11n. **(g)** Peach-YOLO.

**Figure 16:**
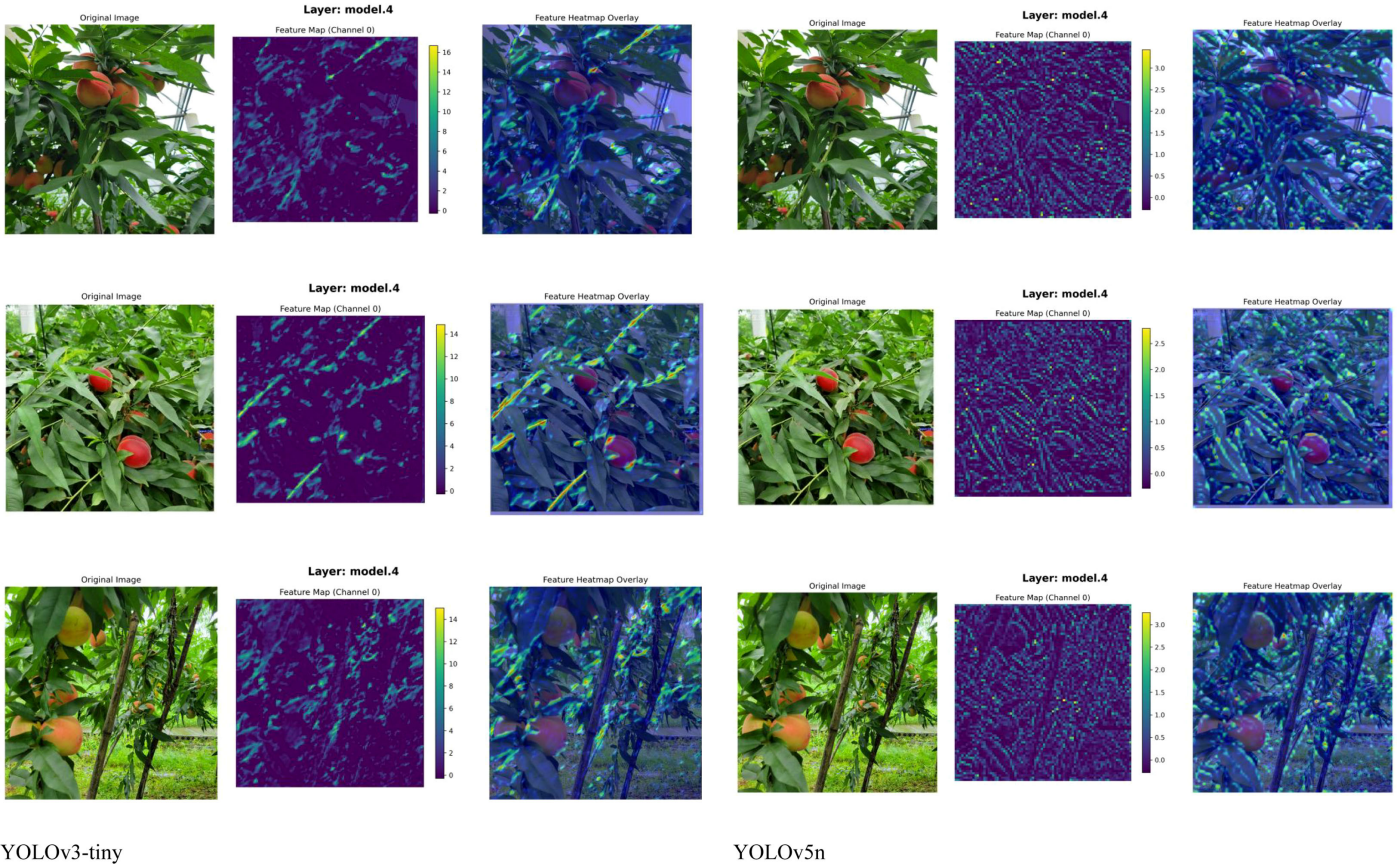
Comparison of heat map results.

**Figure 17:**
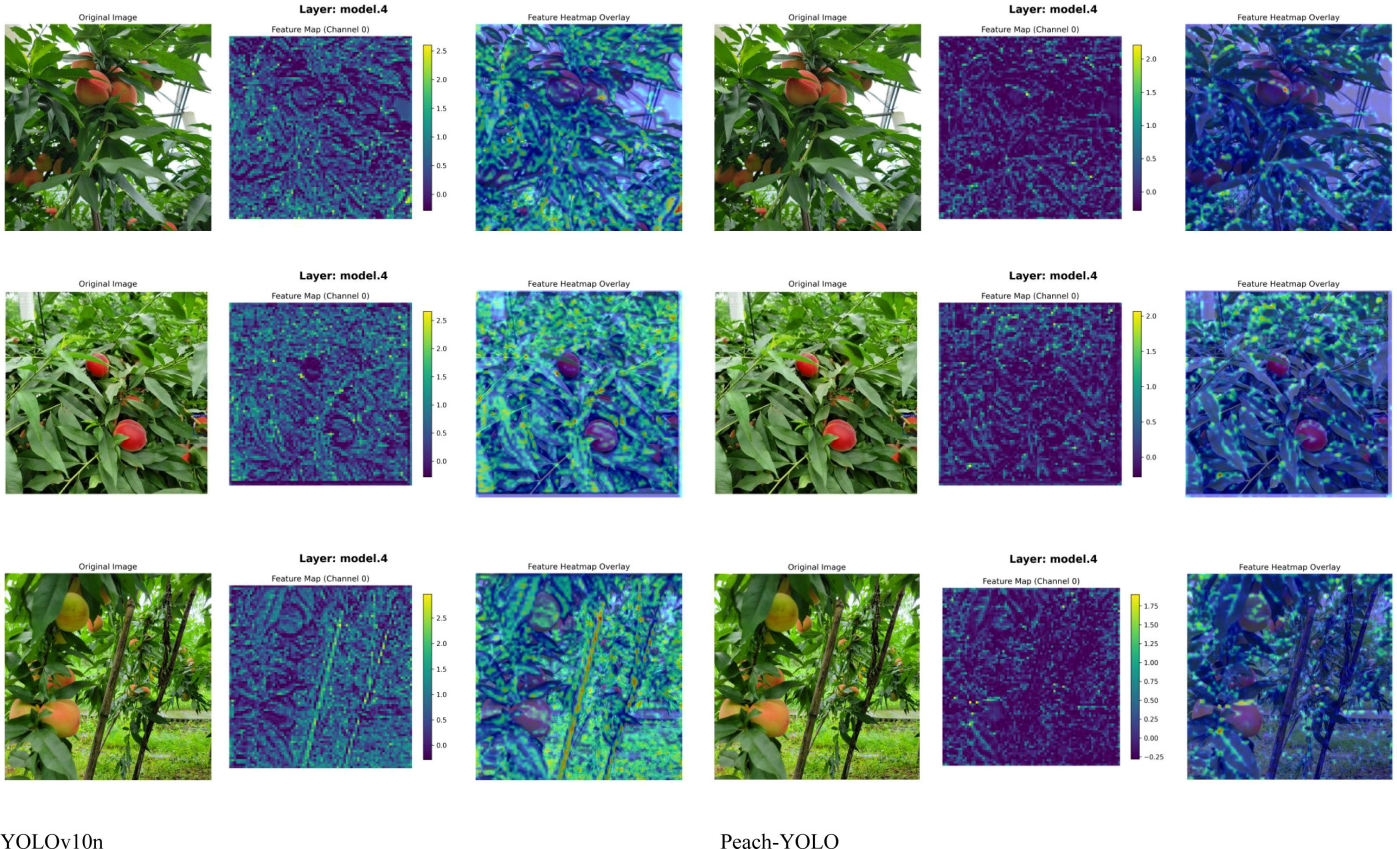
Comparison of feature map results.

To provide a more intuitive comparison of detection performance, the improved model (Peach-YOLO) and various baseline models were evaluated on the test set, with the results shown in **Figures 15**–**17**. The comparison of detection results in **Figure 15** demonstrates that the improved model (g) exhibits a significant reduction in missed detections, duplicate detections, and false detections. It particularly shows robust performance in challenging scenarios involving leaf and branch occlusion as well as small targets. The comparison of heat maps in **Figure 16** indicates that the activation regions of the improved model are more concentrated and precise in focusing on the fruit bodies themselves, effectively suppressing background interference. This validates the attention-enhancing effect of the RFAConv and CAFM modules. The comparison of deep feature maps (C2f module) in **Figure 17** further reveals the feature advantages of the improved model. The corresponding layers from models possessing the C2f structure were selected for comparison (hence, Faster R-CNN, YOLOv7, and YOLOv11n are not included). The results show that the C2f module in the improved model generates clearer feature responses with less noise. This indicates that the CA-HSFPN structure significantly enhances feature discriminability by optimizing gradient flow paths and information fusion methods. The visualization results collectively indicate that through attention enhancement, feature fusion optimization, and loss function improvement, the proposed method enables the model to learn purer and more discriminative feature representations. Consequently, it achieves more accurate and robust peach detection in complex natural environments. This provides an effective technical solution for the vision system of peach-picking robots.

## 4 Conclusions

This study constructed a detection dataset based on images collected from orchard peach trees and proposed an attention-enhanced lightweight model named Peach-YOLO. Through multi-level attention fusion and structural optimization, the model achieved lightweight deployment while improving accuracy. The main conclusions are as follows:

1. Systematic enhancement via attention mechanisms significantly improved the model’s perception and localization accuracy in complex scenes. The introduction of the Receptive-Field Attention Convolution (RFAConv) and the Convolution-Attention Fusion Module (CAFM) into the backbone network effectively strengthened feature extraction in salient fruit regions and suppressed background interference. The neck employs a Coordinate Attention-guided High-Level Screening Feature Pyramid Network (CA-HSFPN), which achieves adaptive recalibration of channel and spatial information and enhances multi-scale semantic fusion capability. Furthermore, the bounding box regression accuracy was optimized using the Shape-IoU loss function. These improvements collectively enhanced the model’s detection performance in occluded, overlapping, and small-target scenarios.
2. A well-designed structure balanced lightweight deployment and real-time performance. While continuously integrating attention modules to boost performance, computational complexity was controlled through structural optimization. The final model size is only 5.0 MB, achieving a real-time inference speed of 115.7 FPS on standard hardware, fully meeting the deployment requirements for orchard robotic systems.
3. Experimental results validated the effectiveness and superiority of the model. On the custom dataset, Peach-YOLO achieved an mAP@0.5 of 82.2%, a precision of 78.9%, and a recall of 76.4%, with significant improvements over the baseline YOLOv8n (increases of 3.0, 3.6, and 4.8 percentage points, respectively). Compared to mainstream models such as YOLOv5n, YOLOv7, YOLOv10n, and Faster R-CNN, Peach-YOLO demonstrated clear advantages in the overall trade-off between accuracy and speed, particularly maintaining low false positive and false negative rates under complex occlusion conditions.

In summary, by integrating attention mechanisms with lightweight design, Peach-YOLO achieves a synergistic improvement in detection accuracy and operational efficiency. This system provides a reliable and easily deployable vision solution for automated peach picking, laying a foundation for subsequent integration and closed-loop experiments of deploying Peach-YOLO into a robotic arm control system, thereby advancing the application of agricultural robotics technology.

## Data Availability

The raw data supporting the conclusions of this article will be made available by the authors, without undue reservation.
